# Materials for pulpotomy in immature permanent teeth: a systematic review and meta-analysis

**DOI:** 10.1186/s12903-019-0917-z

**Published:** 2019-10-23

**Authors:** Yuan Chen, Xinlei Chen, Yali Zhang, Fangjie Zhou, Jiaxin Deng, Jing Zou, Yan Wang

**Affiliations:** 1 State Key Laboratory of Oral Diseases, National Clinical Research Center for Oral Diseases & Department of Pediatric Dentistry, West China Hospital of Stomatology, Sichuan University, Chengdu, 610041 China; 2 Department of Stomatology, The first people’s hospital of Qujing, Qujing, 655000 China

**Keywords:** Pulpotomy, Immature permanent teeth, Pulp exposure, Randomized controlled trials, Systematic reviews

## Abstract

**Background:**

Pulpotomy is one of the most widely used methods in preserving vital pulp in teeth, which is of great significance in achieving continue root formation in immature permanent teeth suffering from dental caries or trauma. The aim of this meta-analysis and systemic review is to synthesize the available evidences to compare different pulpotomy dressing agents for pulpotomy treatment in immature permanent teeth.

**Methods:**

Electronic databases including MEDLINE (via Pubmed), EMBASE, the Cochrane library (CENTRAL) and the clinicaltrials.gov database were searched. The references of all included articles or relevant reviews were cross-checked. Only randomized controlled trials (RCTs) comparing two or more pulp dressing agent in permanent teeth with open apex would be included. Also, the studies should have at least 6 months of follow-up, report clinical and radiographic success in detail and publish in English.

**Results:**

Five RCTs were included for a systematic review, and all of them had a high risk of bias. There is little difference in success rate between mineral trioxide aggregate (MTA) and calcium hydroxide (CH) at 6-month follow-up (risk ratio (RR) 1; 95% confidence interval (CI) 0.94 to 1.06) and 12-month follow-up (RR 1.04; 95% CI 0.96 to 1.13). There is no difference between MTA versus platelet-rich fibrin and MTA versus calcium-enriched mixture (CEM). There is only weak evidence of increased success rate in using MTA and triple antibiotic paste (TAP) rather than abscess remedy.

**Conclusions:**

Based on the present evidence, similar success rates with MTA were found between the dressing agents CH, CEM, RPF and TAP as pulpotomy-dressing agents in the treatment of immature permanent teeth. More high-quality RCTs are needed in this field in future studies.

## Background

Immature permanent teeth, also known as young permanent teeth, are used to describe teeth with incomplete root formation. Immature permanent teeth are prone to caries and trauma which can cause exposure and degeneration of pulp. Pulp degeneration stops root formation, leaving teeth with open apex. It is vital to preserve the pulp vitality otherwise the incompletion of root might result in the fragility of teeth. Pulpotomy is recommended by the American Association of Pediatric Dentistry for the management of pulp exposure in immature permanent teeth to achieve apexogenesis (continued root formation and closure of apex) [1]. In this procedure the partial or coronal pulp tissue is removed in order to eliminate the infected or contaminated pulp and to reach the healthy vital pulp [2].

After pulp tissue removal, pulpotomy dressing agent is applied to the pulp surface, allowing the pulp to heal and the root to form. The selection of agent can influence the success rate of vital-pulp therapy [3]. Mineral trioxide aggregate (MTA) is a universally accepted pulpotomy agent. Its mechanism of action is similar to the traditional pulpotomy agent calcium hydroxide (CH) because MTA releases CH inducing dentine formation when applied to vital pulp [4]. MTA has taken the place of CH as the first choice for pulpotomy recently due to its better capability in apexogenesis, disinfection capabilities, biocompatibility and lack of cytotoxicity [5]. However, it also has its drawbacks, including discoloration of teeth [6], high pH during the procedure [7], high cost and high technical sensitivity [8, 9]. Therefore, alternative choices of pulpotomy dressing agents are being provided to provide more significant induction of dentine formation, higher biocompatibility and better cost-effectiveness. Calcium-enriched mixture (CEM) [10], platelet-rich fibrin (PRF) [11] and antibiotics mixture such as triple antibiotic paste (TAP) are also administered in the treatment of pulpotomy in immature permanent teeth.

As more pulpotomy dressing agents are being introduced in the treatment of pulp exposure caused by caries or trauma, the different treatment outcomes of each agent should be evaluated. Recent meta-analyses have evaluated the outcome of pulpotomy in primary and apex-closed permanent teeth and both analyses showed positive results in the application of pulpotomy [12, 13]. However, the outcome of pulpotomy in immature permanent teeth has not yet been analyzed. Thus, the objective of this systemic review and meta-analysis is to assess the success rate of pulpotomy in immature permanent teeth with carious or traumatic exposed pulp, focusing on the difference between different dressing agents.

## Materials and methods

This systematic review was carried out following the Transparent Reporting of Systematic Reviews and Meta-Analyses (PRISMA) guidelines [14].

### Focused PICO question

Our focused question was based on the Participants, Interventions, Control and Outcomes (PICO) principle: ‘For immature permanent teeth receiving pulpotomy, which medicament was the best choice?’

### Literature search and study selection

An electronic literature search was conducted using MEDLINE (via Pubmed), EMBASE, the Cochrane library (CENTRAL) and the clinicaltrials.gov database between the inception date and October 2018. The following search strategy was adapted for each database search: (pulpotomy OR pulpotomies OR pulp therapy OR pulp treatment OR pulp exposure) AND (permanent OR adult OR secondary) AND (random*), limited in ‘English’.

In order to identify potentially eligible studies, two independent authors screened the titles and abstracts which were derived from the electronic search. The full texts of all candidate studies were further evaluated to identify studies that met all inclusion criteria. To avoid missing any eligible studies, the references of all included articles or relevant reviews were also screened. Agreement between reviewers in the selection procedure was calculated by the Cohen’s kappa statistics, assuming κ = 0.6 as an eligible score. Any discrepancies were resolved by discussion.

### Eligible criteria

Studies were considered eligible if they met the following criteria:

1. Randomized clinical trials (RCTs) comparing two or more pulpotomy medicaments.
2. Pulpotomy was conducted in vital immature permanent teeth with open apex (in human).
3. Having at least 6-month follow-up period.
4. Reporting clinical or radiographic success rates.
5. Describing the criteria of clinical and radiographic success clearly. Clinical success was defined as no pain, no abscess or fistulation, no excessive tooth mobility and no swelling. Radiographic success was considered if the teeth showed no evidence of apical and furcal radiolucency, internal or external root resorption, periodontal ligament widening, or periapical bone destruction.
6. The articles were published in English.

Non-RCTs or RCTs focusing on mature permanent teeth were excluded.

### Data collection and analysis

Two independent authors extracted and managed data from the included studies into a specially designed table (Table 1). When data was incomplete or missing, we tried to contact authors to obtain the missing part. Any differences were resolved by discussion and the accuracy of the data was confirmed by the third author. We treated each tooth or root as units of analysis, and clinical and radiographic success rates were derived in this systematic review using the same criteria: deleting drop-outs and only considering patients who recalled. The success rate was classified as dichotomous data, and we expressed the estimate of effect of an intervention as risk ratios (RRs) together with 95% confidence intervals (CIs). The I^2^ test on the level of α = 0.10 was used to evaluate statistical heterogeneity. When there was statistically significant heterogeneity (I^2^ > 50%), a random-effect model was used to analyze the data; otherwise (I^2^ ≤ 50%), a fixed-effect model was used instead. The statistical significance for the hypothesis test was set at α < 0.05 (two-tailed z tests). The analysis was performed using the Review Manager 5.3 software provided by the Cochrane Collaboration. When meta-analysis could not be performed, the data were summarized qualitatively.

**Table 1:** Characteristics of the included studies

Study	Country	Design	Age (years)	Treatment	Group	Restoration	Analysis unit	Follow-up (months)	Number of analyzed unit	Clinical success rate	Radiographic success rate
Nosrat 2006	Iran	RCT, Parallel	6–10	Full pulpotomy	CEM (*n* = 59) MTA (*n* = 59)	GIC	Root	6	CEM (*n* = 55) MTA (*n* = 55^a^)	CEM (100%) MTA (100%)	CEM (100%) MTA (100%)
								12	CEM (*n* = 57) MTA (*n* = 55^b^)	CEM (100%) MTA (100%)	CEM (100%) MTA (100%)
Keswani 2014	India	RCT, Parallel	6–12	Full pulpotomy	PRF (*n* = 31) MTA (*n* = 31)	Amalgam SSC	Tooth	6	PRF (*n* = 30) MTA (*n* = 29)	PRF (100%) MTA (100%)	PRF (100%) MTA (100%)
								12	PRF (*n* = 29) MTA (*n* = 27)	PRF (100%) MTA (100%)	PRF (100%) MTA (100%)
								24	PRF (*n* = 27) MTA (*n* = 26)	PRF (100%) MTA (100%)	PRF (100%) MTA (100%)
Özgür 2017	Turkey	RCT, Parallel	6–13	Partial pulpotomy	MTA (*n* = 40) CH (*n* = 40)	Composite resin	Tooth	6,12	CH (*n* = 39) MTA (*n* = 40)	MTA (97.5%) CH (97.4%)	MTA (97.5%) CH (97.4%)
								18, 24	CH (n = 39) MTA (*n* = 37)	MTA (97.3%) CH (97.4%)	MTA (97.3%) CH (97.4%)
Eppa 2018	India	RCT, Parallel	6–14	Full pulpotomy	MTA (*n* = 20) TAP (*n* = 20) AR (*n* = 20)	GIC SSC	Tooth	1	MTA (*n* = 20) TAP (*n* = 20) AR (*n* = 20)	MTA (100%) TAP (100%) AR (100%)	MTA (100%) TAP (100%) AR (100%)
								3, 6, 9, 12, 18, 24	MTA (*n* = 20) TAP (*n* = 20) AR (n = 20)	MTA (100%) TAP (100%) AR (80%)	MTA (100%) TAP (100%) AR (80%)
El-Meligy 2006	Egypt	RCT, split-mouth	6–12	Partial pulpotomy	MTA (*n* = 15) CH (*n* = 15)	Amalgam Composite resin	Tooth	3, 6	MTA (*n* = 15) CH (*n* = 15)	MTA (100%) CH (100%)	MTA (100%) CH (100%)
								12	MTA (*n* = 15) CH (*n* = 15)	MTA (100%) CH (86.7%)	MTA (100%) CH (86.7%)

*AR* abscess remedy, *CEM* calcium-enriched mixture cement, *CH* calcium hydroxide, *GIC* glass ionomer cement, *MTA* mineral trioxide aggregate, *PRF* platelet-rich fibrin, *SSC* stainless steel crown, *TAP* triple antibiotic paste

^a^, 2 root was not interpretable; ^b^, 1 root was not interpretable

### Quality assessment

The quality of the included studies was assessed by two independent authors. The authors used the Cochrane risk of bias assessment tool for seven domains. Each domain was divided into three categories: low risk of bias, unclear risk of bias and high risk of bias. The studies were classified as low risk of bias if all domains were evaluated to be of low risk, as moderate risk if one or more domains were evaluated to be of unknown risk, or as high risk if any of the domains were evaluated as high risk. The inter-examiner agreement was analyzed by kappa coefficient, and any disagreements were resolved by discussion.

## Results

### Results of the literature

**Fig. 1:**
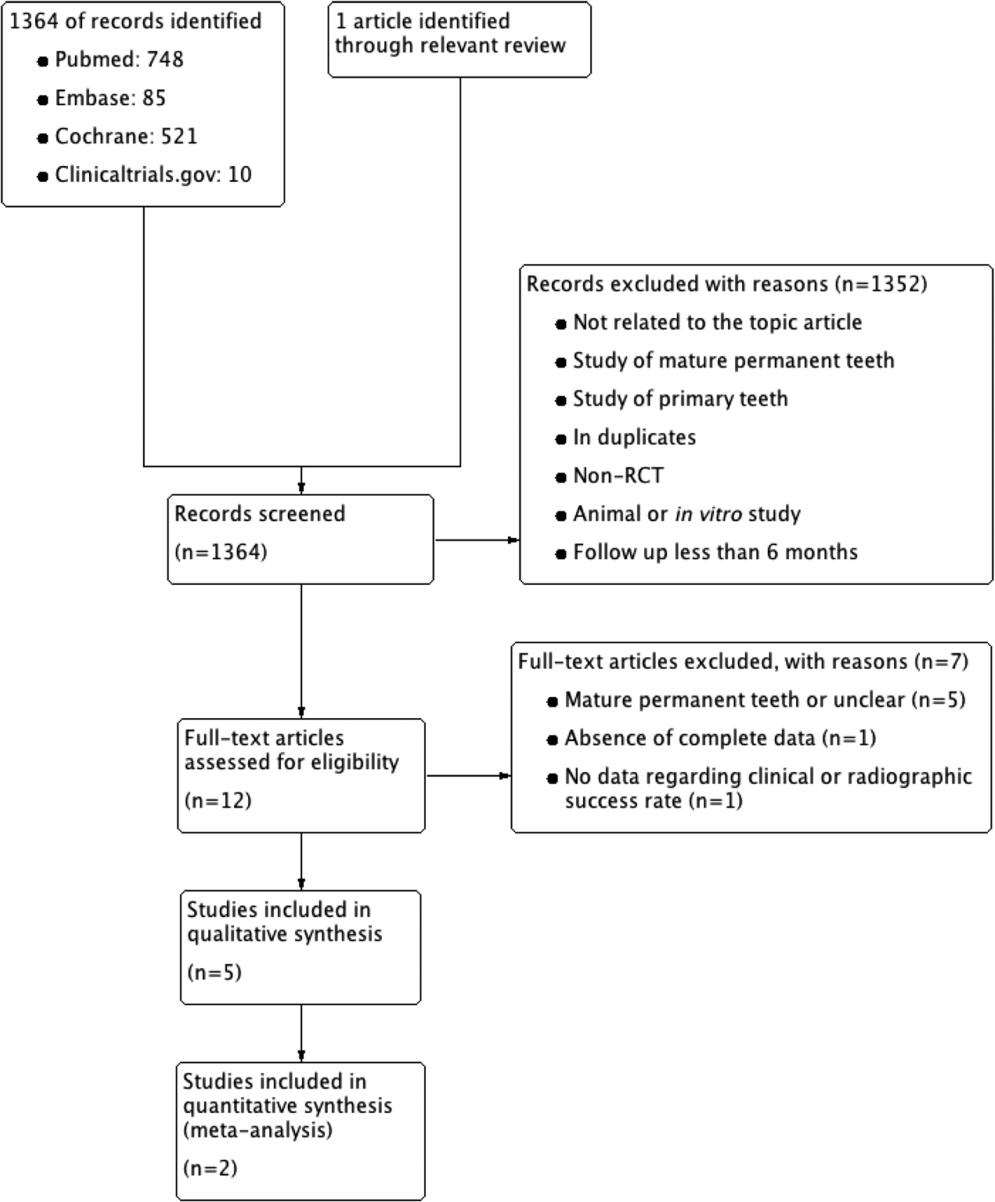
Search retrieval flow diagram

A total of 1365 articles were retrieved from the databases during the search process (Fig. 1). After screening titles and abstracts, a full-text assessment of 12 articles was conducted by 2 independent investigators (inter-reviewer agreement, kappa = 0.91). Finally, five studies [15–19] were finally selected, two of which were chosen for meta-analysis at 6 and 12-month periods respectively [17, 18]. The study selection process is presented as a flow chart in Fig. 1, and the characteristics of each included study are summarized in Table 1.

The five included studies were published between 2006 and 2018, two of which were conducted in India [16, 19], one in Iran [15], one in Turkey [18] and one in Egypt [17]. Of the five studies, four were parallel trials and the remaining study was a split-mouth trial. Of the included studies, two compared MTA with CH [17, 18], one compared CEM with MTA [15], one compared PRF with MTA [16], and one compared MTA, TAP and abscess remedy [19]. Four studies with 232 teeth used each tooth as a unit of analysis [16–19], while the other one with 118 roots used each root as a unit [15]. Only one study [17] included traumatized teeth and carious teeth, all of the other four studies only included carious teeth [15, 16, 18, 19]. The sample sizes varied from 30 to 80 teeth. The mean age of patients was described in the five studies and ranged from 6 to14 years old. Follow-up periods also varied in these studies, ranging from 12 to 24 months.

### Quality analysis

**Fig. 2:**
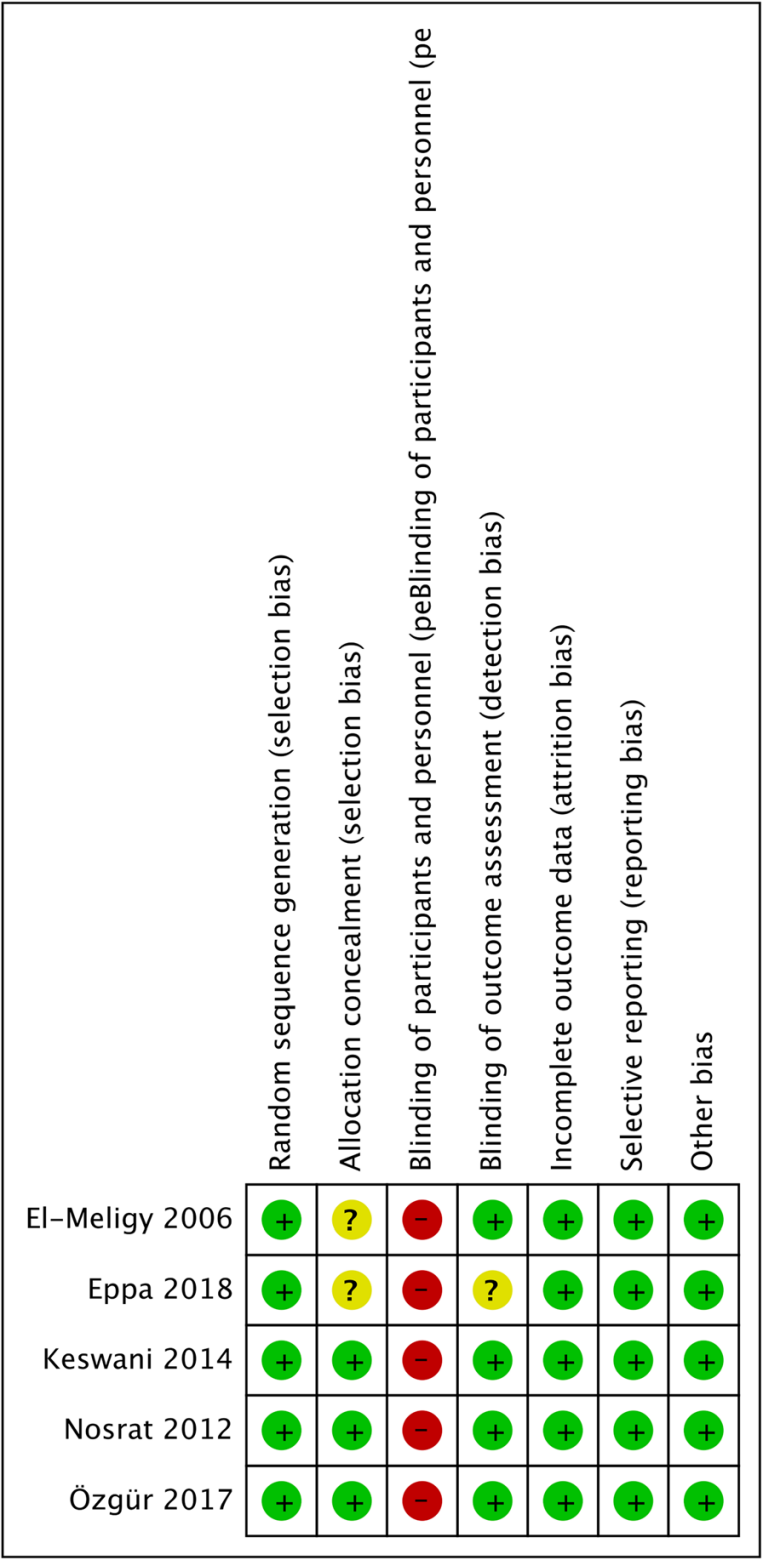
Risk of bias assessment

The quality of the included studies was assessed using the Cochrane risk of bias assessment tool (Fig. 2). All of the included studies were evaluated to have a high risk of bias due to the types of interventions that do not permit the blinding of the operators. Four studies had a low risk of detection bias [15–18], as they reported the blinding of the investigator, whereas the remaining study was assessed as unclear risk of detection bias as they did not mention whether the investigator was blinded [19]. Three of the included trials clearly described randomized methods [15, 16, 18], but the other two did not provide details as to which study selection method was used [17, 19]. The agreement between the reviewers was 0.93.

### Primary outcomes- clinical and radiographic success rate

#### Comparison 1: MTA versus CH

Both El-Meligy 2006 and Özgür 2017 compared MTA with CH. El-Meligy 2006 (40 teeth in the MTA group and 40 teeth in the CH group) described outcomes in 3, 6 and 12-month follow-ups and Özgür 2017 (15 teeth in the MTA group and 15 teeth in the CH group) reported 6, 12, 18 and 24-month follow-up outcomes [17, 18]. There was no significant clinical heterogeneity between the two studies, so we pooled their data at 6 and 12 months.

##### 6-month outcome

**Fig. 3:**
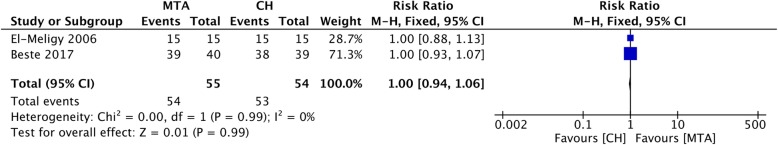
Forest plot of comparison: mineral trioxide aggregate (MTA) versus calcium hydroxide (CH) at 6 months

We pooled the two studies with 55 teeth in the MTA group and 54 teeth in the CH group (Özgür 2017 reported 1 tooth dropped out at this period in the CH group). Fifty-four teeth in the MTA group were clinically and radiographically successful and 53 teeth in the CH group. There was no statistically significant difference in neither the clinical nor radiographic success rate between MTA and CH at 6 months of follow-up (risk ratio (RR) 1; 95% confidence interval (CI) 0.94 to 1.06)) (Fig. 3).

##### 12-month outcome

**Fig. 4:**
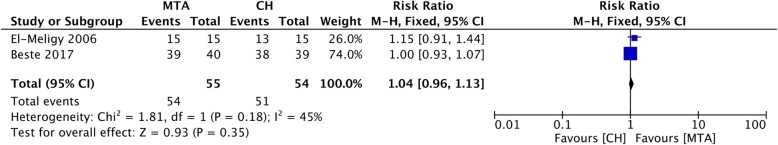
Forest plot of comparison: mineral trioxide aggregate (MTA) versus calcium hydroxide (CH) at 12 months

At 12-month evaluation, the number of teeth was 55 for MTA group and 54 for CH group. It also showed no difference between MTA and CH on the rate of clinical and radiographic success (RR 1.04; 95% CI 0.96 to 1.13) (Fig. 4). Fifty-four teeth were treated successfully in the MTA group and 53 teeth were treated successfully, in the CH group.

##### Other timepoints

In the MTA group, Özgür 2017 showed a 97.3% clinical and radiographic success rate for both the 18 and 24-month follow up periods (36 out of 37, 3 teeth dropped out at 18 months). While in the CH group, it was a 97.4% success rate at the same two follow up periods (38 out of 39, 1 tooth dropped out at 6 months). There was no significant difference between the two groups (*P* > 0.05). El-Meligy 2006 reported 100% clinically and radiographical success rates at 3 months in the two groups (15 out of 15 for both groups).

#### Comparison 2: MTA versus CEM

Only Nosrat 2012 compared MTA with CEM (59 roots in MTA, 59 roots in CEM). In the MTA group 2 roots were not interpretable and 4 roots dropped out 6 months. Also at 6 months in the CEM group 4 roots dropped out (15). After 12 months, 1 root was not interpretable and 4 roots dropped out in the MTA group, while 2 roots dropped out in the CEM group. The authors showed a 100% clinical and radiographical success rate for both groups at 6 and 12 follow-up periods (6 months: 53 roots out of 53 in the MTA group; 55 roots out of 55 in the CEM; 12 months: 54 roots out of 54 in the MTA and 57 roots out of 57 in the CEM group). There were no statistical differences between the two groups in terms of clinical and radiographic success at all follow-up periods (*P* > 0.05).

#### Comparison 3: MTA versus PRF

Only Keswani 2014 compared MTA with PRF (31 teeth in both the MTA group, and PRF group) [16]. At 6 months in the MTA and PRF group, 2 and 1 teeth dropped out respectively; at 12 months, another 2 and 1 teeth dropped out respectively and at 24 months, 1 and 2 teeth dropped out respectively. The authors revealed all follow-up appointments all teeth were successful, clinically and radiographically (6 months: in the MTA group, 29 teeth out of 29 and in the PRF group 30 teeth out of 30; and at 12 months, in the MTA group, 27 teeth out of 27 and in the PRF group, 29 teeth out of 29; at 24 months: 26 teeth out of 26 in the MTA group and 27 teeth out of 27 in the PRF group).

#### Comparison 4: MTA versus TAP versus abscess remedy

Only Eppa 2018 compared MTA with triple antibiotic paste and abscess remedy at 1, 3, 6, 9, 12, 18, 24 months [19]. No cases dropped out during any of the follow-up periods. Only 4 out of 20 cases in the abscess remedy group failed. These failed cases were reported at 3 months. The clinical and radiographic success rates were statistically significant between the two groups at 3, 6, 9, 12, 18 and 24 months (*P* < 0.05).

### Secondary outcomes-adverse events

Özgür 2017 reported 2 teeth in the MTA group at 18 months and 1 tooth in the CH group at 24 months with marginal discoloration of the restorations, but no significant correlation was found between marginal integrity failures and the clinical/radiographic failures (*P* > 0.05) [18]. El-Meligy 2006 found calcific metamorphosis in 2 teeth treated with CH and 4 teeth treated with MTA [17].

## Discussion

The goal of this study was to systematically review the available information on pulpotomy dressing agents for treating immature permanent teeth, which would help paediatric dentists to make treatment choices on their clinics based on the best scientific evidence available. To the author’s knowledge, this is the first systematic review focusing on the difference between the various agents in treating immature permanent teeth with pulp exposed to caries or trauma. We found five randomized clinical trials which compared four of the following treatment comparisons in terms of clinical and radiographical results: MTA versus CH, MTA versus CEM, MTA versus PRF, MTA versus TAP and abscess remedy. Based on these studies, similar success rates with MTA were found between the dressing agents CH, CEM, RPF and TAP as pulpotomy-dressing agents in the treatment of immature permanent teeth. However, most of the outcomes were based on single studies. Additional research comprised of larger well-conducted randomized trials comparing one pulpotomy-dressing agent with another used in immature permanent teeth is needed to reach a definitive conclusion.

### Quality of studies

All the five included studies had high risk of bias. The study of Eppa 2018 [19] did not mention the blindness of investigators. Also the studies of Eppa 2018 [19] and El-Meligy 2006 [17] did not describe the randomization method in detail. Reporting bias and other bias were not observed in the included studies. All the studies were assessed to have high risk of bias in performation bias because they did not mention the concealment of group to operators in their study. Considering that the various agents would present in different appearance concerning the color, physical form and preparing procedure, it is not practical to blind the operators. Performance bias, under this circumstance, seems unavoidable. The same problem also exists in other studies related to different agents for pulpotomy [20]. Researchers could try to minimize bias through isolating the investigator from the operating process, which might make the ivestigator quantify the effect of the interventions more objectively. To conclude, there are limited publications in this field and bias might affect the accuracy of conclusions drawn from the aforementioned studies. Clinicians could benefit from more well-organized randomized controlled trials in future.

### Different dressing agents on success rate

The challenge in the management of immature permanent teeth with pulp exposed to caries or trauma is to maintain the pulp vitality as well as achieve continued root formation. Factors concerning the success rate of pulpotomy include accurate diagnosis before treatment [21], well-handled isolation [22], thorough disinfection [23], rigorous restoration using glass ionomer cement (GIC) and resin or amalgam and different pulpotomy-dressing agents. Materials used in pulpotomy dressing usually affect the success rate of pulpotomy. An ideal pulpotomy dressing material should be biocompatible, capable of hard tissue formation, have disinfectant properties and lack of cytotoxity. Although technology is developing and more new materials have emerged, there is not yet one single recommended gold standard pulpotomy dressing.

El-Meligy 2006 [17] and Özgür 2017 [18] both compared the outcome between MTA and CH; their criteria for success were both symptom free, absence of radiographic abnormality and continued root formation. A meta-analysis was performed to evaluate the success rate at 6 months and 12 months. Results were consistent in the 6-month and 12-month follow-ups that no significant difference was observed between the use of MTA and CH. Based on the current limited evidence, MTA and CH had similar outcome of pulpotomy in immature permanent teeth, which was consistent with previous research in permanent teeth with closed apices [12] but different with the conclusion drawn by previous systematic review in primary molars [13]. MTA is considered superior to CH in clinical and radiographic aspects in primary molar as a result of better biocompatibility of MTA [24]. The differences in these conclusions might have occurred due to the theory that immature permanent teeth have richer blood supply and have greater resistance to the infection and contamination, which improves the success rate of immature permanent teeth and hence, reduces the difference between MTA and CH [25]. This conclusion may be biased, owing to the lack of high quality studies and limited number of study subjects.

MTA, as the most utilized pulpotomy-dressing agent in young permanent teeth nowadays and it was the research focus of all included studies. Similar treatment outcomes were observed between MTA, PRF, CEM and TAP and only abscess remedy presented a less satisfying outcome of treatment, with more teeth showing pain and tenderness along with periapical radiolucency [19]. Abscess remedy is described to be an updated radiopaque bacterial paste consists of cresol, polyoxymethylene, cinnamon oil and excipient [19]. Research on the use of abscess in pulpotomy is scanty. Further research on abscess remedy in vitro and in vivo should be conducted to understand the effectiveness of abscess remedy. TAP is another bacterial paste that was analyzed in Eppa’s research [19]. TAP consists of a mixture of three different antibiotics: ciprofloxacin, metronidazole and minocycline and it shows its superiority to abscess remedy in the success rate of pulpotomy in immature permanent teeth. If the pulpotomy treatment is about to succeed, microbiota should be properly reduced, abscess remedy and TAP both function as antibacterial agents to improve the treatment outcome [26].

Platelet-rich fibrin is a second-generation platelet concentration with autologous nature that equips it with higher biocompatibility than synthetic materials such as MTA [27]. It has a physical structure favorable of healing, when activated, signaling molecule were released to control the recruitment of cells, morphogenesis and process of inflammation [28, 29]. With its consistent success rate with MTA and better biocompatibility than MTA, PRF would be a good substitute for MTA and CH in the treatment of pulp exposure in immature permanent teeth. However, it also comes with its limitation that it requires certain amount of fresh blood and requires a special machine to prepare the agent.

Calcium-enriched mixture is water-based cement first introduced to endodontic treatment by Asgary in 2006 [30]. It is a mixture of different calcium compounds including, calcium oxide, calcium phosphate, etc. [31]. It shows similarities with MTA in its sealing ability [32], biocompatibility [33] and the potential to induce hard tissue [34]. The result of this study agrees with the aforementioned theories. This agent also shows its advantages in less tooth-discoloration [35] and stronger antibacterial ability [36] than MTA, thus it can also be considered as a good substitute for MTA.

The adverse events reported in the above studies was calcific metamorphosis and it was observed in both the MTA and CH group [17, 18]. Calcific metamorphosis is a common finding in pulpotomized teeth, it is a sign of pulpal vitality and it is a result of vigorous odontoblastic activity. Both materials are known to prompt hard tissue and hence it is not surprising, and it is reported that the incidence of pulp canal obliteration is 4 to 24% in tooth after dental trauma [37]. Although it is not considered as a criteria for clinical success or failure, the calcification of canal chamber can increase the difficulty in future treatment and lead to the facture or perforation when trying to locate canals. As a common adverse event for MTA, crown discoloration caused by the oxidation of heavy metal oxides (ie. iron or bismuth) [38] was not reported in the included studies, which might be a result of limited follow-up time or the insufficient number of subjects. Marginal discoloration was reported, suggesting the microleakage of restoration in marginal area, which might open an access for bacteria. Microbial leakage will cause infection and hence affect the success of pulp treatment [39]. However, no correlation was observed between the failures of integrity and clinical/radiographic failures in the included study. The authors assume that the resin degrade due to the contact of saliva might lead to the marginal discoloration and a long-term follow up is needed to elucidate the outcomes.

### Limitations of study

The most obvious limitation of this review is the small number of included studies and that the small sample size of all the included studies. Most of the comparisons were based on single studies. All of the above would influence the accuracy of conclusion.

Also, data pooling in our research is poor. For one thing, one of the included studies use every single root as an unit for outcome assessment while others use one tooth as a single unit, this is an obstacle for data synthesis. We also found that the criteria for radiographic success differed among the included studies, which also hindered the data synthesis. All of the included studies agreed that 1) absence of perioapical radiolucency, 2) no external or internal resoption, 3) no widened periodontal ligament, 4) no signs of destruction to the lamina dura, and 5) continued root growth are esstienal factors for radiographic success. However, there remains conflicts in whether the complete apical closure should be a requisite factor. Norsrat et al. regarded complete apical closure as radiographic success criteria [15], Eppa et al. mentioned it in their criteria but did not reported the rate of complete root closure in their results [19]. According to the guideline of American Association of Endodontists (AAE) and AAPD, the objective of pulpotomy is to prevent the clinical symptoms, avoid root resorption and breakdown of periodontal tissue as well as to radiographically observe continued root growth, neither of them mentioned that complete apical closure must be achieved [2, 40]. It is vital to unify the criteria of success for pulpotomy in immature permanent teeth.

### Directions for future study

Based on the limitation that mentioned above, future researches would benefit from the following strategies: Firstly, a more detailed methodology including randomization method and blinding method should be developed in future studies to enhance the quality of studies. Secondly, unification of criteria for clinical/radiographic outcome assessment should be established. Thirdly, using root or tooth as a single unit for outcome assessment should be further discussed. More high-quality studies in this filed are expected in the near future to address the clinical question: When undergoing pulpotomy for immature permanent teeth, which medicament should we choose?

## Conclusion

To conclude, pulpotomy is an effective way in achieving apexogenesis in immature permanent teeth with pulp exposed to dental caries and trauma. However, based on the present limited evidence, similar success rates with MTA were found between the dressing agents CH, CEM, RPF and TAP as pulpotomy-dressing agents in the treatment of immature permanent teeth, and there is insufficient evidence to draw any conclusion as to the benefits of one material over another. Peadiatic dentists may consider cost-effectiveness when choosing pulpotomy-dressing agents in clinics. More high-quality randomized controlled trials evaluating the effect of different pulpotomy dressing agents on immature permanent teeth with pulp exposure to dental caries or trauma are required.

## Data Availability

All data generated and analyzed in this study are included within the article or available from the authors.

## Abbreviations

CEM: Calcium-enriched mixture
CH: Calcium hydroxide
Cl: Confidence interval
I^2^: Higgins index
MTA: Mineral trioxide aggregate
RR: Risk ratio
TAP: Triple antibiotic paste
